# Oxidative Damage in Korean Medicine

**DOI:** 10.3390/antiox11030600

**Published:** 2022-03-21

**Authors:** Gunhyuk Park

**Affiliations:** Herbal Medicine Resources Research Center, Korea Institute of Oriental Medicine, 111 Geonjae-ro, Naju-si 58245, Korea; gpark@kiom.re.kr or parkgunhyuk@gmail.com; Tel.: +82-61-338-7112

Korean medicine originated in ancient and prehistoric times, as evidenced by the discovery of stone and bone needles in the North Hamgyeong Province, dating back to 3000 BC [1,2]. According to the records of Korea’s founding myth, Gojoseon tells the story of tigers and bears who wanted to be reborn as humans by eating mugwort and garlic [3,4]. Poetry written around the time of the Three Kingdoms describes mugwort and garlic as “foods”, indicating that medicinal herbs were used as treatments [4]. At that time, various herbal medicines were used for pain relief and treatment of wounds, along with a knowledge of healthy foods.

It is known that Korean medicine developed during the Three Kingdoms Period, Goryeo Dynasty, and Unified Silla Period, and flourished more during the Joseon Dynasty [2]. In particular, in the 15th century, efforts were made to promote the values of research and development of various Korean medicines, with the publication of highly valued texts such as Hyangyakjipseongbang, the Medical Encyclopedia, a summary of the king’s history, and confidential agreements [5,6]. Since then, many medical texts have been published, including Donguibogam written by Heo Jun, a Korean medical doctor who further integrated Korean and Chinese medicine at the time. Ever since its publication, it has had such a great influence on medicine in China, Japan, and Vietnam that in 2009, UNESCO decided to register Donguibogam as a UNESCO World Heritage Site [7,8]. At this juncture, Korean medicine was recognized as an important academic asset for researchers worldwide. Many researchers worldwide have applied this medical basis as a treatment strategy for various diseases. In particular, many researchers have made great efforts to demonstrate the antioxidant effects of these medicinal herbs. This is because aging-related and other degenerative diseases are characterized by increased oxidative stress. The past few decades have witnessed a heightened interest in antioxidants, which prevent aging and other various degenerative diseases [9,10]. Reactive oxygen species (ROS) are highly reactive molecules that are continuously produced in biological reactions, with major active oxygen-forming skeletons and excess active oxygen neutralized by biological defense mechanisms, including enzymes, vitamins, and a variety of antioxidants [10,11]. When these defense mechanisms are depleted or free radicals are in excess, the possibility of damage to biological polymers, such as proteins, DNA, and lipids, due to oxidative stress increases [10,11]. The damage caused reflects the severity of chronic diseases, and many researchers are making great efforts to treat diseases using antioxidants [10].

Through this Special Issue, we aim to promote the possibility of leading a richer human life by using traditional Korean medicine-based modern medical experimental methods to prevent or treat diseases with excellent antioxidant effects, systematic theorization, and value as industrial materials, such as health functional foods and cosmetics. Thus, this Special Issue of antioxidants collected original research papers on the complex biomedical effects of pro- and antioxidants that affect aging-related disease control. Due to the tough selection process for submitted papers carried out by the editorial office of the journal, the majority of submitted papers were not published in this Special Issue, but we hope that further progress in the field will include their contributions.

Kang et al. reported that Peucedanum japonicum and its active components protect the ocular surface against urban particulate matter-induced oxidation [12]. After a screening of herbal extracts, Peucedanum japonicum Thunberg extracts were found to promote the recovery rate of wounded corneas and efficiently improve the wound healing and migration activity of corneal epithelial cells. The major components were defined as chlorogenic acid, neochlorogenic acid, and cryptochlorogenic acid, which were individually effective on the migration activity and synergistically enhanced this effect. Thus, corneal abrasion wound recovery after urban particulate matter exposure improved after Peucedanum japonicum Thunberg treatment, and its active components were identified, providing an important basis for developing therapeutics for ocular surface damage, focusing on interleukin-6.

Ji et al. reported that P. grandiflorum crude saponin attenuates amyloid beta-induced neurotoxicity by inhibiting intracellular reactive oxygen species in 5XFAD mice [13]. In particular, P. grandiflorum crude saponin downregulated nuclear factor-κB-mediated inflammatory signaling and apoptosis signaling via nuclear factor erythroid 2–related factor action. In addition, P. grandiflorum crude saponin ameliorates amyloid beta-mediated pathologies, leading to Alzheimer’s disease-associated cognitive decline. Thus, P. grandiflorum crude saponin is a resource for developing therapeutics for Alzheimer’s disease.

S-Y Kim et al. demonstrated the effects of Mentha arvensis essential oil on lipopolysaccharide-induced inflammatory stimulation in RAW 264.7 macrophages and HaCaT keratinocytes [14]. This research team confirmed the possibility of applying these findings to an AD model. They verified that there was significant atopic dermatitis inhibitory effect bought about by the suppression of the extracellular signal-regulated kinase/nuclear factor-κB signaling pathway.

Eom et al. showed that naringin acted in a concentration-dependent and voltage-insensitive manner, reversibly inhibited the interaction with transient receptor potential cation channel subfamily V member 1 at the cellular and molecular levels, and had an IC_50_ value of 33.33 μM. Naringin only inhibited inward peak current without affecting inward peak current [15]. They cross-checked the results from TEVC and docking experiments and found that D471 and N628 of transient receptor potential cation channel subfamily V member 1 were involved in binding with naringin. These results are thought to be an important basis for developing a treatment for degenerative disease using naringin.

Lee et al. demonstrated that Hydrangea macrophylla and its active compound, thunberginol C, improved chronic restraint stress or corticosterone-induced anxiety behavior activity in mice [16]. These extracts also inhibited corticosterone and tumor necrosis factor-α levels in the blood. They also prevented oxidative responses such as glutathione peroxidase, glutathione reductase activity, and superoxide dismutase in the hippocampus.

Yang et al. reported that Insamgobonhwan attenuated RAS-selective lethal 3-induced ferroptosis and suppressed amyloid-β-induced cognitive impairment [17]. In particular, insamgobonhwan inhibited cell death and lipid peroxidation, which were increased by RAS-selective lethal 3 administration. In addition, Insamgobonhwan restored the expression of ferroptosis marker proteins such as glutathione peroxidase 4 and heme oxygenase 1, which were altered by RAS-selective lethal 3. This effect was confirmed in mice with Alzheimer’s disease treated with amyloid beta, which points towards the possibility of dementia treatment in the future.

Many researchers continue their attempts to reveal ancient Korean oriental medical treatments, understand them and prove their efficacy from a modern perspective. I hope that readers will be interested in aging-related diseases and that this Special Issue will attract the interest of the scientific community, thereby supporting further investigations that lead to the discovery of novel strategies for the use of therapeutic medicinal herbs in the field of aging.
